# Fn14 Deficiency Ameliorates Anti-dsDNA IgG-Induced Glomerular Damage in SCID Mice

**DOI:** 10.1155/2018/1256379

**Published:** 2018-12-16

**Authors:** Jiawen Wu, Xiaoyun Min, Li Wang, Jie Yang, Ping Wang, Xingyin Liu, Yumin Xia

**Affiliations:** ^1^Department of Dermatology, The Second Affiliated Hospital, School of Medicine, Xi'an Jiaotong University, Xi'an 710004, China; ^2^Core Research Laboratory, The Second Affiliated Hospital, School of Medicine, Xi'an Jiaotong University, Xi'an 710004, China; ^3^Department of Nephrology, The Second Affiliated Hospital, School of Medicine, Xi'an Jiaotong University, Xi'an 710004, China; ^4^Department of Nephrology, Tangdu Hospital, Air Force Military Medical University, Xi'an 710038, China; ^5^Department of Immunology and Microbiology, Wannan Medical College, Wuhu 241002, China; ^6^Department of Pathogenic Biology, Nanjing Medical University, Nanjing 211166, China

## Abstract

Many studies have demonstrated that anti-dsDNA IgG is closely associated with lupus nephritis. Recently, it was found that activation of the fibroblast growth factor-inducible 14 (Fn14) signaling pathway damages glomerular filtration barrier in MRL/lpr lupus-prone mice. However, MRL/lpr mice have high titers of serum autoantibodies other than anti-dsDNA IgG. The aim of this study was to further explore the effect of Fn14 deficiency on anti-dsDNA IgG-induced glomerular damage in severe combined immunodeficiency (SCID) mice that have no endogenous IgG. Fn14 deficiency was generated in SCID mice. The murine hybridoma cells producing control IgG or anti-dsDNA IgG were intraperitoneally injected into mice. In two weeks, the urine, serum, and kidney tissue samples were harvested from mice at sacrifice. It showed that the injection of anti-dsDNA IgG, but not control IgG hybridoma cells, induced proteinuria and glomerular damage in SCID mice. Between the wild-type (WT) and knockout (KO) mice injected with anti-dsDNA IgG hybridoma cells, the latter showed a decrease in both proteinuria and glomerular IgG deposition. The histopathological changes, inflammatory cell infiltration, and proinflammatory cytokine production were also attenuated in the kidneys of the Fn14-KO mice upon anti-dsDNA IgG injection. Therefore, Fn14 deficiency effectively protects SCID mice from anti-dsDNA IgG-induced glomerular damage.

## 1. Introduction

Lupus nephritis (LN) is one of the most common complications occurring in the internal organs of patients with systemic lupus erythematosus (SLE). Although the precise mechanism of LN remains unclear, many studies strongly suggested that anti-double-stranded (ds) DNA IgG is pivotal in the pathogenesis of LN [1–3]. Serum levels of anti-dsDNA IgG increase in patients with LN and fluctuate with flares of lupus disease [4]. Anti-dsDNA IgG binds to circulating nuclear antigens and forms immune complexes, which further deposit in the glomerular basement membrane. Alternatively, anti-dsDNA IgG recognizes multiple renal self-antigens through cross-reaction, such as histone, heparan sulfate, laminin, collagen IV, alpha-actinin, and annexin II [3, 5–9]. Such cross-reaction between anti-dsDNA IgG and renal components leads to direct immune complex formation in local tissues or even IgG penetration into renal resident cells [6, 7]. SCID mice that receive intraperitoneal injection of anti-dsDNA IgG hybridoma cells exhibit renal anti-dsDNA IgG deposition, urine albumin, and microstructural changes in the glomeruli [5]. Anti-dsDNA IgG induces fibronectin secretion of renal tubular epithelial cells and myofibroblast-like phenotype of mesangial cells [10, 11], which are early-stage events of renal fibrosis, a histopathologic feature associated with poor outcomes of LN [12]. Additionally, anti-dsDNA IgG contributes to renal fibrosis through selective inhibition of the suppressor of cytokine signaling 1 signals [13]. Inhibition of anti-dsDNA IgG production may ameliorate renal damage in LN [2, 14]. Obviously, anti-dsDNA IgG is nephritogenic and participates in the pathophysiological processes of LN.

Tumor necrosis factor-like weak inducer of apoptosis (TWEAK) is a key regulator of proinflammatory cytokines and chemokines. TWEAK acts in the local tissues through engaging its sole receptor fibroblast growth factor-inducible 14 (Fn14). The expression of Fn14 is relatively lower in normal cells, but increases significantly in local tissues under inflammatory conditions [15–19]. The TWEAK/Fn14 signaling pathway is involved in various autoimmune disorders, such as polymyositis or dermatomyositis, systemic sclerosis, bullous pemphigoid, and psoriasis [17, 18, 20, 21]. Moreover, TWEAK/Fn14 signals play an important role in the pathogenesis of lupus erythematosus. TWEAK/Fn14 activation contributes to the damage of the skin, kidney, and brain [22–24]. Fn14 is upregulated in the glomeruli of mice with acute kidney injury, spontaneous LN, or antibody-mediated nephritis, and Fn14 deficiency or neutralizing anti-TWEAK antibody attenuates renal injuries in these models [25–27]. TWEAK/Fn14 interaction can modulate gene expression, cytokine production, and cell cycle (proliferation or apoptosis) of renal resident cells [23, 26, 27]. Therefore, TWEAK/Fn14 activation is prominent in tissue injuries of SLE, including LN.

An interesting phenomenon is that Fn14 deficiency attenuates LN in the MRL/lpr lupus-prone mice, but not affecting serum titers of anti-dsDNA antibodies [27]. Fn14 deficiency significantly reduces proteinuria, glomerular and tubulointerstitial diseases, leukocyte infiltration, and even glomerular IgG deposition in these mice. Nevertheless, the serum levels of total IgG and anti-dsDNA IgG as well as the numbers and distribution of splenocytes are comparable between the Fn14-deficient and wild-type (WT) mice [27]. Moreover, both *in vivo* and *in vitro* experiments showed that Fn14 deficiency preserves the integrity of the renal filtration barrier [27]. These findings suggest that TWEAK/Fn14 activation is pivotal in the pathogenesis of LN.

However, current studies only provide indirect evidence indicating different roles of anti-dsDNA IgG and TWEAK/Fn14 signals in the progression of LN, and there are no results interpreting the relationship between them in the kidneys. Moreover, MRL/lpr mice have high titers of serum autoantibodies other than anti-dsDNA IgG, which may also contribute to the progression of LN [28]. Therefore, the role of TWEAK/Fn14 signals in the nephritogenicity of anti-dsDNA IgG should be elucidated exclusively in the model with anti-dsDNA IgG. The purpose of this study was aimed at revealing the effect of Fn14 deficiency on glomerular injuries of mice that only generate anti-dsDNA IgG.

## 2. Materials and Methods

### 2.1. Generation of Fn14-Deficient Mice

Fn14 deficiency was generated in severe combined immunodeficiency (SCID) mice (NOD.CB17-Prkdcscid/NcrCrl strain; Charles River Laboratories, Wilmington, MA, USA) by using a clustered regularly interspaced short palindromic repeat (CRISPR)/CRISPR-associated (Cas) 9 approach [29]. In brief, Cas9 mRNA and small guide RNA were synthesized *in vitro* and then injected into one-cell stage embryos of SCID mice. The F0 generation mice were identified for genotypes by polymerase chain reaction (PCR) and hybridized with other SCID mice. Finally, the Fn14−/− homozygote mice were selected for the next reproduction. Both CRISPR/Cas9 engineering and reproductive experiments were conducted in Shanghai Biomodel Organism Science & Technology Development Company (Shanghai, China). The strategy for CRISPR/Cas9 engineering is detailed in Figure S1.

### 2.2. Injection of Hybridoma Cells

Murine hybridoma cells of anti-dsDNA IgG (WJ31 clone, IgG2a) or control IgG (WJ77 clone, IgG2a) were purchased from Jieqing Biotech (Wuhan, China) [13]. The Fn14-knockout (KO) and WT mice aged 8 weeks received priming of pristine, followed by intraperitoneal injection of 1 × 10^7^ hybridoma cells per mouse [5]. There were eight mice in each group. Urine, blood, and kidney tissue samples were collected 2 weeks after the injection. The levels of serum IgG (total IgG or IgG isotypes) were measured by enzyme-linked immunosorbent assay as described [27]. Animal experiments were approved by the hospital research ethics committee.

### 2.3. Immunohistochemistry and Immunofluorescence

Immunohistochemistry was performed as described previously [13]. Paraffin sections were incubated with rabbit primary antibodies to Fn14, Ki-67, platelet-derived growth factor subunit B (PDGFB), CD3, Iba-1, or phospho-epidermal growth factor receptor (pEGFR) (2 *μ*g/ml; Abcam, Cambridge, MA, USA). The secondary antibody was polymer-horseradish peroxidase-conjugated goat anti-rabbit IgG (1 *μ*g/ml; DAKO, Glostrup, Denmark). 3,3′-Diaminobenzidine chromogen substrate (DAKO) was used for color development. Some sections were routinely stained with trichrome or hematoxylin and eosin. By following histological scoring systems [27], the stains were scored by two renal pathologists blinded to the mouse grouping. The pathological changes (by hematoxylin and eosin staining), fibrotic changes (by trichrome or PDGFB staining), and inflammatory cell infiltration (by CD3 or Iba-1 staining) in glomeruli were each scored from 0 to 4 (0, absent; 1, mild; 2, mild–moderate; 3, moderate; and 4, severe) by two pathologists blind to the grouping. Glomerular proliferation was quantitated by counting the number of Ki-67-positive cells in 20 glomeruli of each section.

IgG deposition was detected by immunofluorescence in frozen sections [13]. Sections were incubated with fluorescein isothiocyanate-conjugated goat anti-mouse IgG isotypes (2 *μ*g/ml; Southern Biotech, Birmingham, AL, USA). A digital fluorescent microscope (Carl Zeiss, Jena, Germany) was used for viewing fluorescence in the sections.

### 2.4. Transmission Electron Microscopy and Immunogold Staining

As described previously [5], kidney tissue sections were incubated in saturated sodium metaperiodate solution. After blocking with 1% bovine serum albumin in phosphate-buffered saline, sections were incubated with gold-labeled donkey anti-mouse IgG (Electron Microscopy Sciences, Hatfield, PA, USA). Finally, sections were postfixed in 2% glutaraldehyde solution and examined under an electron microscope (JEOL, Peabody, MA, USA).

### 2.5. Real-Time Quantitative PCR

Total RNA was extracted from fresh kidney tissues of mice and processed for cDNA by reverse transcription. SYBR Green Master Mix (Invitrogen, Grand Island, NY, USA) was used as a fluorescent dye. PCR was carried out on the 7900HT Fast PCR System (Applied Biosystems, Carlsbad, CA, USA). The sequences of primers (Jieqing Biotech) are detailed in Table S1.

### 2.6. Western Blotting

Fresh tissues were extracted for protein lysates with the addition of protease inhibitor cocktail (Thermo Scientific, Waltham, MA, USA). Proteins were transferred onto polyvinylidene difluoride membranes, followed by incubation with rabbit IgG targeting Fn14 or pEGFR (2 *μ*g/ml; Abcam). Biotinylated goat anti-rabbit IgG (Southern Biotech) was the secondary antibody (2 *μ*g/ml). After incubation with horseradish peroxidase-streptavidin, an ECL solution kit (Thermo Scientific, Waltham, MA, USA) was used for signal development. The intensities of bands were quantitated by ImageJ 1.61u software (National Institutes of Health, Bethesda, MD, USA) and normalized to the values of *β*-actin bands accordingly.

### 2.7. Measurement of Proteinuria, Urine Creatinine and Monocyte Chemotactic Protein 1 (MCP-1), and Blood Urea Nitrogen (BUN)

The levels of urine albumin were determined by an enzyme-linked immunosorbent assay kit (Bethyl Laboratories, Montgomery, TX, USA). Urine creatinine and BUN were determined by using commercial kits (BioAssay Systems Inc., Hayward, CA, USA). MCP-1 was determined with a commercial enzyme-linked immunosorbent assay kit (R&D Systems, Minneapolis, MN, USA). The protocols were provided by the manufacturers.

### 2.8. Statistical Analysis

All data were expressed as means ± standard error of the mean. The STATA 10.0 software package (StataCorp, College Station, TX) was used for analyzing these data. Analysis of variance was used for comparing more than two groups. A two-tailed unpaired Student *t*-test was then used for comparison of the two groups. Differences were considered significant at *p* < 0.05.

## 3. Results

### 3.1. Fn14 Deficiency Significantly Reduces Proteinuria in SCID Mice

**Figure 1:**
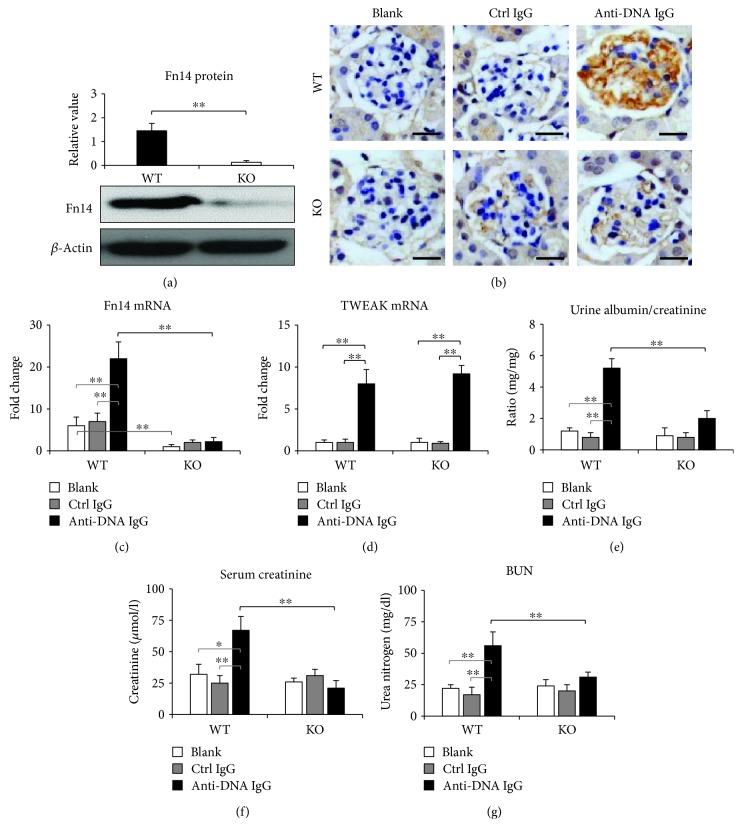
Fn14 deficiency reduces proteinuria in SCID mice. (a) Fn14 protein was determined in the kidneys by Western blotting. (b) IgG deposition was detected in glomeruli by immunohistochemistry. (c) The mRNA expression levels of Fn14 were determined in the kidneys by real-time PCR. (d) Similarly, the mRNA expression levels of TWEAK were also determined. (e–g) The levels of urine albumin, serum creatinine, and blood urea nitrogen (BUN) were determined in mice at sacrifice. There were eight mice in each group. Representative images are shown. Fn14: fibroblast growth factor-inducible 14; TWEAK: tumor necrosis factor-like weak inducer of apoptosis. Scale bar = 50 *μ*m. ^∗^ *p* < 0.05 and ^∗∗^ *p* < 0.01.

The expression levels of Fn14 were determined in the kidneys of SCID mice. By Western blotting and real-time PCR, the WT mice exhibited higher protein and mRNA expression of Fn14 when compared with the KO mice (Figure 1). Moreover, both immunohistochemistry and real-time PCR revealed that the anti-dsDNA IgG hybridoma cell-injected WT mice had higher Fn14 expression in glomeruli when compared with the KO mice (Figures 1(a) and 1(b)). There were no significant differences in Fn14 expression between the WT and KO mice that received control IgG hybridoma cells or those that had no injection. The renal mRNA expression of TWEAK was comparable between the anti-dsDNA IgG-injected WT mice and KO mice although both of them had higher levels than the two control groups accordingly (Figure 1(d)). Furthermore, renal function was evaluated in these mice, revealing that the anti-dsDNA IgG hybridoma cell-injected WT mice had higher levels of urine albumin, serum creatinine, and BUN than the other mice, and these levels decreased significantly in the KO mice (Figures 1(d)–1(f)).

### 3.2. Glomerular IgG Deposition Decreases in Fn14-Deficient SCID Mice

**Figure 2:**
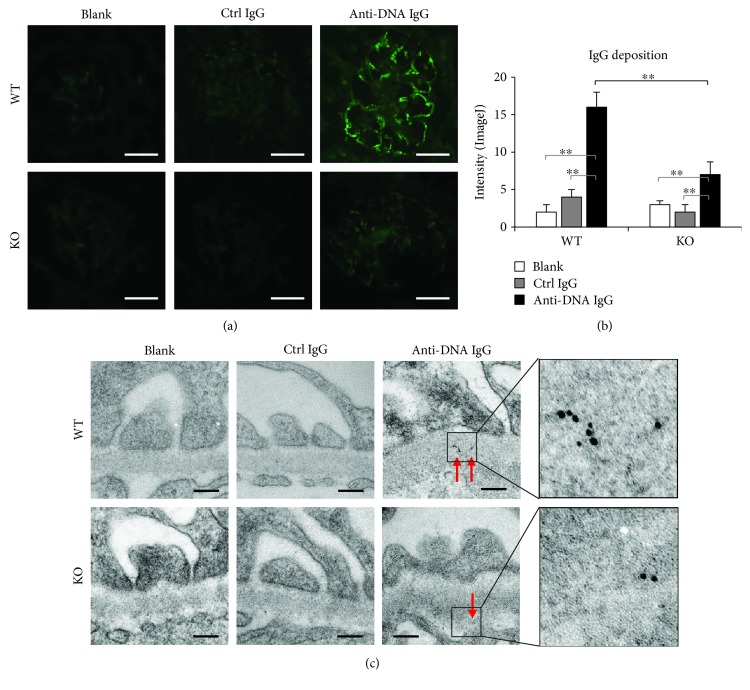
Glomerular IgG deposition is attenuated in Fn14-deficient SCID mice. (a) IgG deposition was detected in glomeruli by immunofluorescence. Scale bar = 50 *μ*m. (b) Fluorescent intensities of glomeruli were quantitated by ImageJ software. (c) By immunogold staining and transmission electron microscopy, IgG deposits were detected in glomeruli. Gold particles were indicated by red arrows. Scale bar = 200 nm. There were eight mice in each group. Representative images are shown. ^∗∗^ *p* < 0.01.

Because TWEAK/Fn14 activation affects renal IgG deposition in lupus-prone mice [27], we further examined IgG deposition in the glomeruli of these mice. By immunofluorescence, the anti-dsDNA IgG hybridoma cell-injected WT mice had the strongest fluorescent intensity in glomeruli (Figures 2(a) and 2(b)). Also, IgG deposits were detected by immunogold staining and transmission electron microscopy, showing that IgG deposition was the most prominent in the glomerular basement membrane of the anti-dsDNA IgG hybridoma cell-injected WT mice (Figure 2(c)). Fn14 deficiency partially reduced both fluorescence and gold particles of anti-dsDNA IgG in glomeruli. There were no differences in IgG deposition between the blank and the control IgG groups (Figure 2). Interestingly, the serum levels of total IgG or anti-dsDNA IgG were comparable between the WT and KO mice that were injected with either control IgG or anti-dsDNA IgG hybridoma cells or as blank controls (Figure S2). Serum IgG1, IgG2b, and IgG3 were not detectable in these mice (data not shown). By immunofluorescence, we also found no deposition of these three isotypes in glomeruli (data not shown).

### 3.3. Histopathological Changes Are Attenuated in Fn14-Deficient Kidneys

**Figure 3:**
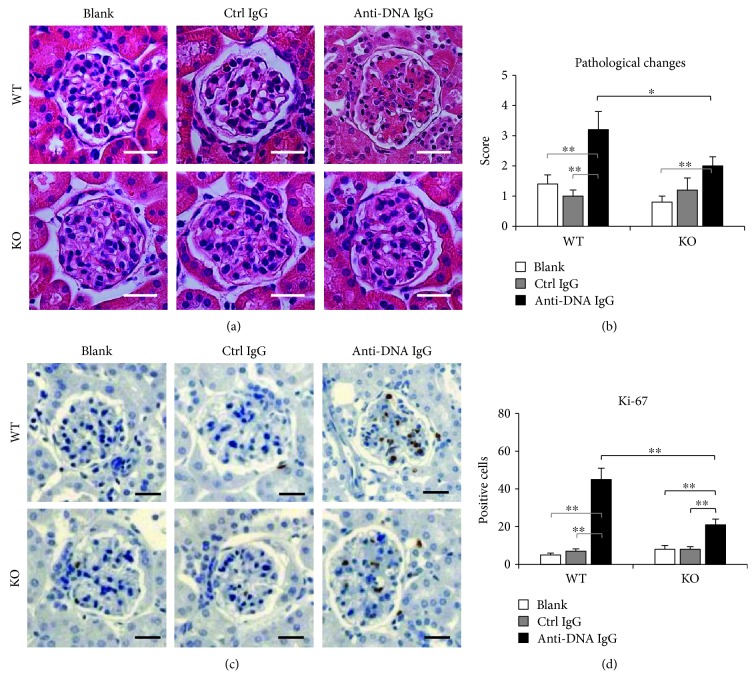
Histopathological changes are ameliorated in Fn14-deficient glomeruli. (a) Kidney sections were stained by hematoxylin and eosin. (b) The hematoxylin and eosin-stained sections were scored for glomerular damage. (c) By immunohistochemistry, Ki-67-positive cells were detected in glomeruli. (d) The Ki-67-positive cells were counted in glomerular areas. There were eight mice in each group. Representative images are shown. Fn14: fibroblast growth factor-inducible 14. Scale bar = 50 *μ*m. ^∗^ *p* < 0.05 and ^∗∗^ *p* < 0.01.

To elucidate the effect of Fn14 deficiency on microstructure of the glomeruli, we evaluated the histopathological changes in these mice. The hematoxylin and eosin-stained kidney sections were scored, showing that the anti-dsDNA IgG hybridoma cell-injected WT mice had the most severe damage in glomeruli (Figures 3(a) and 3(b)). Such histopathological changes were attenuated in the anti-dsDNA IgG hybridoma cell-injected KO mice, which still had a higher score than the KO mice receiving no IgG injection (Figures 3(a) and 3(b)). Glomerular proliferation also reflects the inflammatory severity in LN [26, 27]. Hence, Ki-67 staining was performed with kidney sections. The results showed that the Ki-67-positive cells lessened in the KO mice when compared with the WT mice, both were injected anti-dsDNA IgG hybridoma cells (Figures 3(c) and 3(d)).

**Figure 4:**
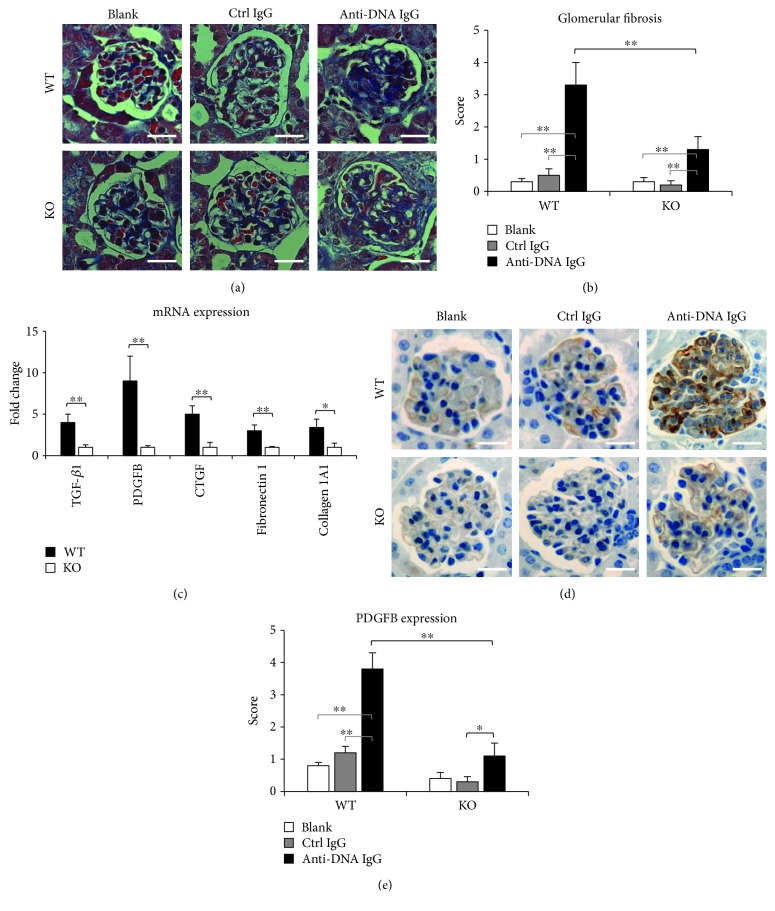
Renal fibrosis is reduced in Fn14-deficient kidneys. (a) Kidney sections were stained by trichrome. (b) The trichrome-stained sections were scored for glomerular fibrosis. (c) By real-time PCR, the mRNA levels of transforming growth factor-*β* (TGF-*β*), platelet-derived growth factor subunit B (PDGFB), connective tissue growth factor (CTGF), fibronectin 1, and collagen 1A1 were determined in kidneys of anti-dsDNA IgG hybridoma cell-injected mice. (d) By immunohistochemistry, PDGFB expression was detected in glomeruli. (e) The PDGFB-stained sections were scored semiquantitatively. There were eight mice in each group. Representative images are shown. Fn14: fibroblast growth factor-inducible 14. Scale bar = 50 *μ*m. ^∗^ *p* < 0.05 and ^∗∗^ *p* < 0.01.

Renal fibrosis is one of the histopathologic features associated with poor outcomes of LN [12] and correlates with the nephritogenicity of anti-dsDNA IgG [13]. We examined glomerular fibrosis in SCID mice by trichrome staining, which showed that the score of tissue fibrosis was the highest in the anti-dsDNA IgG hybridoma cell-injected WT mice but decreased partially in the KO mice receiving the same treatment (Figures 4(a) and 4(b)). Similarly, the mRNA expression levels of transforming growth factor-*β* (TGF-*β*), PDGFB, connective tissue growth factor (CTGF), fibronectin 1, and collagen 1A1 increased in the WT mice but decreased in the KO mice upon injection of anti-dsDNA IgG hybridoma cells (Figure 4(c)). The alteration of PDGFB expression was further confirmed in kidneys by immunohistochemistry (Figures 4(d) and 4(e)).

### 3.4. Less Inflammatory Infiltrate in the Kidneys of Fn14-Deficient Mice

**Figure 5:**
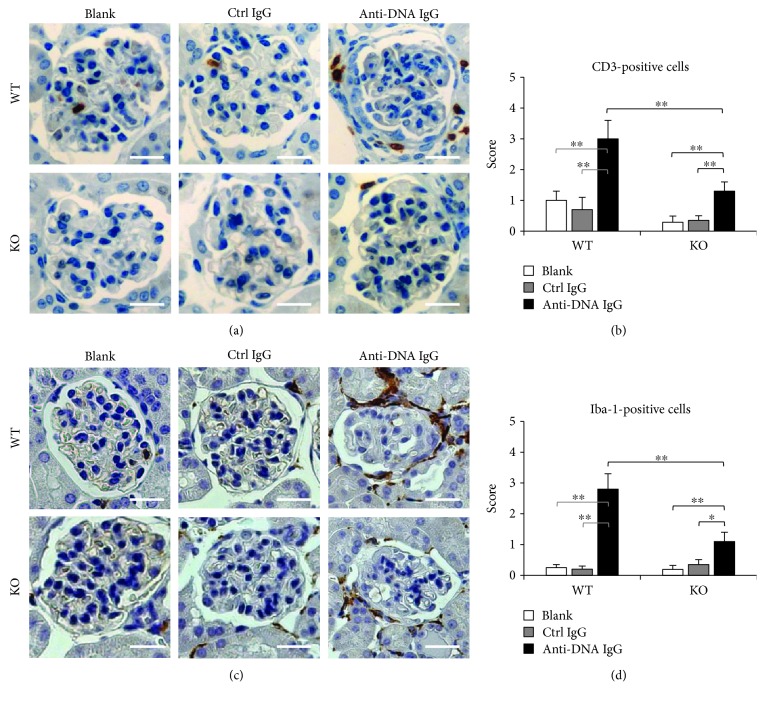
Infiltration of inflammatory cells decreases in glomeruli of Fn14-deficient mice. SCID mice were intraperitoneally injected with hybridoma cells producing control IgG or anti-dsDNA IgG. (a) By immunohistochemistry, CD3-positive cells were detected in glomeruli. (b) The CD3-stained sections were scored by semiquantitative systems. (c) Iba-1-positive cells were also detected by immunohistochemistry. (d) The Iba-1-stained sections were scored semiquantitatively. There were eight mice in each group. Representative images are shown. Fn14: fibroblast growth factor-inducible 14. Scale bar = 50 *μ*m. ^∗^ *p* < 0.05 and ^∗∗^ *p* < 0.01.

Glomerular infiltration of macrophages was evaluated by immunohistochemical staining for CD3- or Iba-1-positive cells. The results showed that injection of anti-dsDNA IgG induced prominent infiltration of these cells in the glomeruli or periglomerular regions of WT mice (Figure 5). However, Fn14 KO in the anti-dsDNA IgG hybridoma cell-injected mice led to remarkable amelioration of inflammatory cell infiltration (Figure 5). By semiquantitative scoring, the anti-dsDNA IgG hybridoma cell-injected WT mice showed significant increase in CD3- or Iba-1-positive cells, which was tempered upon Fn14 deficiency (Figures 5(b) and 5(d)).

### 3.5. Proinflammatory Cytokines Are Downregulated in Fn14-Deficient Kidneys

**Figure 6:**
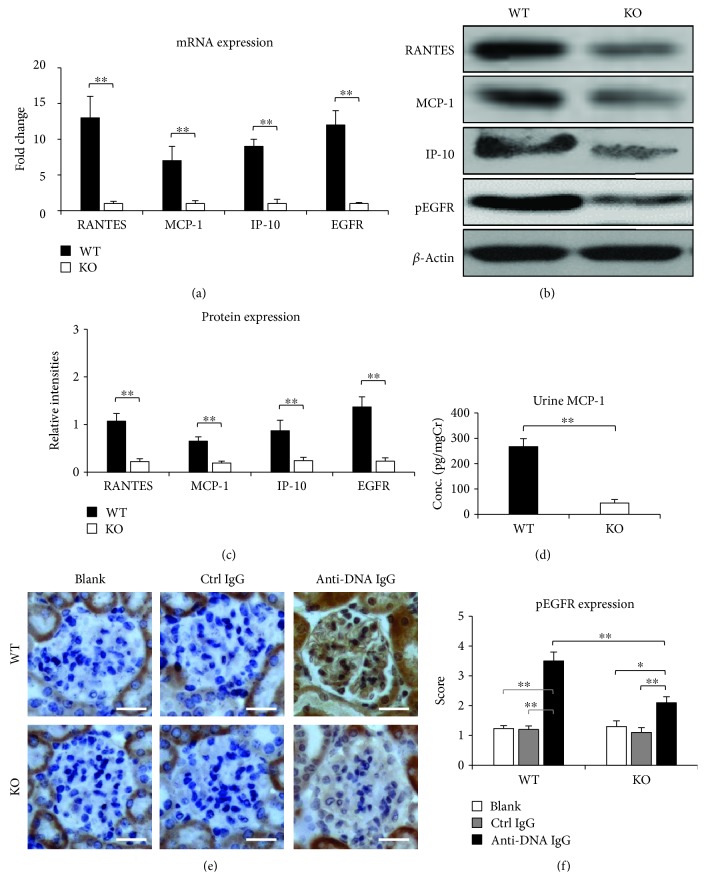
Proinflammatory cytokines and pEGFR are downregulated in Fn14-deficient kidneys. Both WT and KO mice were injected with anti-dsDNA IgG hybridoma cells. (a) By real-time PCR, the mRNA levels of regulated on activation, normal T cell expressed and secreted (RANTES), monocyte chemotactic protein 1 (MCP-1), interferon gamma-induced protein 10 (IP-10), and epidermal growth factor receptor (EGFR) were determined in kidneys. (b) By Western blotting, the proteins of RANTES, MCP-1, IP-10, and phospho-EGFR (pEGFR) were determined in the kidney lysates. (c) The intensities of blots were measured by ImageJ software. (d) Urine MCP-1 levels were determined in different groups. (e) Glomerular pEGFR expression was also detected by immunohistochemistry. (f) The pEGFR-stained sections were scored semiquantitatively. There were eight mice in each group. Representative images are shown. Fn14: fibroblast growth factor-inducible 14. Scale bar = 50 *μ*m. ^∗^ *p* < 0.05 and ^∗∗^ *p* < 0.01.

Proinflammatory cytokines in the kidneys were assessed by real-time PCR. It showed that the mRNA and protein expression levels of regulated on activation, normal T cell expressed and secreted (RANTES), MCP-1, and interferon gamma-induced protein 10 (IP-10) were higher in the WT mice when compared with the KO mice, both of which received injection of anti-dsDNA IgG hybridoma cells (Figures 6(a)–6(c)). In accordance, urine levels of MCP-1 were lower in the KO mice (Figure 6(d)).

Interestingly, EGFR, a transmembrane protein that can be transactivated by TWEAK and contributes to renal disease progression especially fibrosis [30], was also less expressed in the kidneys of the KO mice after injection of anti-dsDNA IgG hybridoma cells (Figures 6(a)–6(c)). There were no significant differences in both proinflammatory cytokines and EGFR between the blank and control IgG hybridoma cell-injected mice. By immunohistochemistry and semiquantitative scoring, the anti-dsDNA IgG hybridoma cell-injected WT mice showed significant increase in glomerular pEGFR expression, which was attenuated upon Fn14 deficiency (Figures 6(e) and 6(f)).

## 4. Discussion

In this study, we demonstrated that anti-dsDNA IgG upregulates TWEAK and Fn14 expression in kidneys and induces prominent glomerular damage in the WT SCID mice. However, Fn14 deficiency significantly reduces proteinuria as well as glomerular IgG deposition in anti-dsDNA IgG hybridoma cell-injected mice. Accordingly, the histopathological changes and inflammatory cell infiltration are attenuated in the kidneys of these Fn14-deficient mice. Furthermore, the expression of proinflammatory cytokines and EGFR is downregulated in Fn14-deficient kidneys. Therefore, Fn14 deficiency effectively ameliorates anti-dsDNA IgG-induced glomerular damage in SCID mice.

Previous studies demonstrated that intraperitoneal injection of anti-dsDNA IgG hybridoma cells induces LN-like renal damage in SCID mice, and Fn14 deficiency attenuates LN in MRL/lpr mice without affecting serum IgG levels [5, 27]. Our results confirmed such nephritogenicity of anti-dsDNA IgG as well as the protective effect of Fn14 deficiency in SCID mice. Because MRL/lpr mice have multiple autoantibodies in sera, which may contribute to renal damage [27], Fn14 deficiency was generated in SCID mice, which have no detectable IgG because of V(D)J recombination impairment [5]. It showed that anti-dsDNA IgG upregulates both Fn14 and TWEAK expression in the kidneys of the WT mice. The downstream proinflammatory cytokines, including RANTES, MCP-1, and IP-10, also increase in these kidneys. These results supported the finding that TWEAK/Fn14 signals activate upon anti-dsDNA IgG deposition in the glomeruli. On the other hand, the Fn14-KO mice showed less glomerular IgG deposition though their serum IgG levels remained the same as those of the WT mice after injection of anti-dsDNA IgG hybridoma cells. Hence, renal deposition of anti-dsDNA IgG correlates closely with local TWEAK/Fn14 activation. There were no differences in circulating the anti-dsDNA IgG level between the two strains though the KO mice had less glomerular IgG deposition. Such discrepancy might be due to persistent production of IgG by intraperitoneal hybridoma cells, which could conceal an actual difference.

Although SCID mice are characterized by the absence of functional T cells and B cells, their kidneys may be infiltrated by macrophages under inflammation [31]. In fact, macrophages are one of the main resources of soluble TWEAK in inflammatory tissues [18]. In this study, macrophages (Iba-1-positive) infiltrated the kidneys of SCID mice after injection of anti-dsDNA IgG hybridoma cells, accompanied by an increase in TWEAK mRNA expression. Anti-dsDNA IgG injection induced less glomerular infiltration of T cells (CD3-positive) in Fn14-KO mice, confirming an inhibitive effect of Fn14 deficiency on T cell recruitment. The expression of Fn14 was significantly elevated in the kidneys of SCID mice upon anti-dsDNA IgG injection. Accordingly, the TWEAK/Fn14 signaling-regulated downstream cytokines, including RANTES, MCP-1, and IP-10, increase in these kidneys. Obviously, immunodeficiency in SCID mice does not block the TWEAK/Fn14 pathway, which is actually activated in anti-dsDNA IgG-induced renal damage.

Renal fibrosis is one of the final outcomes of patients with LN [12]. It has been known that anti-dsDNA IgG participates in the processes of renal fibrosis through blocking the suppressor of cytokine signaling 1 signals and inducing fibronectin secretion or myofibroblast-like phenotype of renal resident cells [10, 11, 13]. TWEAK/Fn14 signaling is also deeply involved in the inflammation-related fibrosis of tissues, including the liver, heart, lung, and kidney [23, 32–35]. TWEAK/Fn14 activation promotes kidney fibrosis involving Ras-dependent proliferation of renal fibroblasts [36]. So, anti-dsDNA IgG deposition and TWEAK/Fn14 activation may cooperate in facilitating renal fibrosis of LN. Our results showed that glomerular fibrosis is apparent in SCID mice after injection of anti-dsDNA IgG hybridoma cells. Moreover, the mRNA expression levels of fibrotic markers, such as TGF-*β*, PDGFB, CTGF, fibronectin 1, and collagen 1A1, increase significantly in kidneys of these mice. However, both glomerular fibrotic scores and fibrotic marker expression decrease upon Fn14 deficiency. These findings not only support the finding that anti-dsDNA IgG contributes to renal fibrosis of LN but also affirm that TWEAK/Fn14 inhibition blocks such effect of anti-dsDNA IgG on kidneys.

RANTES, MCP-1, and IP-10 are TWEAK-induced cytokines that trigger cellular and tissular inflammatory responses [16, 17, 22, 24, 26]. These cytokines are upregulated in kidneys with inflammation and fibrosis and attract inflammatory cells, such as monocytes, T lymphocytes, and neutrophils [37, 38]. Monitoring of these cytokines may help follow up the progression of renal diseases [39, 40]. Our results showed that the mRNA expression levels of RANTES, MCP-1, and IP-10 were higher in the WT mice when compared with the Fn14-KO mice, both of which received injection of anti-dsDNA IgG hybridoma cells. Also, urine MCP-1 level decreased in the KO mice. Therefore, TWEAK/Fn14 activation is more prominent in the WT mice, and Fn14 deficiency protects mice from anti-dsDNA IgG-induced renal inflammation. We speculate that Fn14 inhibition induces less production of these cytokines, thus maintaining the integrity of the glomerular filtration barrier. The rest of the findings such as histological changes as well as inflammatory cell infiltration are secondary to both TWEAK/Fn14 inhibition and decreased IgG deposition.

EGFR is a transmembrane protein that activates through engaging its ligands including epidermal growth factor and transforming growth factor-*α* [41]. Recent studies demonstrated that Fn14 upregulation correlates with EGFR phosphorylation (activation) in tumor cells [42, 43]. Moreover, TWEAK/Fn14 interaction can directly induce phosphorylation of EGFR, which mediates TWEAK-induced proinflammatory factor upregulation and inflammatory cell infiltration in kidneys [30]. Furthermore, TWEAK/Fn14 signaling induces cell proliferation and renal fibrosis through activating the EGFR pathway [30, 44]. In this study, we found that renal expression of EGFR correlates positively with TWEAK/Fn14 activation in SCID mice. This phenomenon also reflects that the TWEAK/Fn14 pathway mediates renal inflammation and fibrosis in anti-dsDNA IgG-treated mice.

## 5. Conclusions

Based on our findings, we conclude that the TWEAK and Fn14 upregulation and glomerular damage are prominent in anti-dsDNA IgG hybridoma cell-injected SCID mice. Fn14 deficiency effectively protects SCID mice from renal damage through reducing glomerular IgG deposition and local inflammatory responses. In future studies, exogenous inhibitory approaches should be developed to suppress the TWEAK/Fn14 pathway in a murine model of LN.

## Data Availability

The data used to support the findings of this study are available from the corresponding author upon request.

## Supplementary Materials

Supplementary Materials Table S1: sequences of primers. Figure S1: the strategy for generating Fn14 knockout mice by the CRISPR/Cas9 method. Figure S2: the serum levels of IgG are comparable between the WT and KO mice.
