# Supplementary material for: Fn14 Deficiency Ameliorates Anti-dsDNA IgG-Induced Glomerular Damage in SCID Mice
Source: J Immunol Res. 2018 Dec 16;2018:1256379. doi: 10.1155/2018/1256379 (PMC6311848; doi:10.1155/2018/1256379)
Supplement: Supplementary Materials — Table S1: sequences of primers. Figure S1: the strategy for generating Fn14 knockout mice by the CRISPR/Cas9 method. Figure S2: the serum levels of IgG are comparable between the WT and KO mice. [file 1256379.f1.pdf]

Table S1: Sequences of primers

Oligonucleotide	Sequence
Collagen 1A1 primers	Forward: 5′-CCTAATGCTGCCTTTTCTGC-3′<br>Reverse: 5′-ATGTCCCAGCAGGATTTGAG-3′
CTGF primers	Forward: 5′-CCTGGTCCAGACCACAGAGT-3′<br>Reverse: 5′-GACAGGCTTGGCGATTTTAG-3′
EGFR primers	Forward: 5′-GTGATCCAAGCTGTCCCAAT-3′<br>Reverse: 5′-CTTGGAACTTTTGGCAGACC-3′
Fibronectin 1 primers	Forward: 5′-AAGACCATACCTGCCGAATG-3′<br>Reverse: 5′-CAACTGGTTGGCATGAAATG-3′
Fn14 primers	Forward: 5′-CTAGTTTCCTGGTCTGGAGAAGATG-3′<br>Reverse: 5′-CCCTCTCCACCAGTCTCCTCTA-3′
IP-10 primers	Forward: 5′-GGATGGCTGTCCTAGCTCTG-3′<br>Reverse: 5′-CCTTGGGAAGATGGTGGTTA-3′
MCP-1 primers	Forward: 5′-GAATCACAAGCAGCCAGTGT-3′<br>Reverse: 5′-GGGAACTTCAGGGGGAAATA-3′
PDGFB primers	Forward: 5′-CCCACAGTGGCTTTTCATTT-3′<br>Reverse: 5′-GTGGAGGAGCAGACTGAAGG-3′
RANTES primers	Forward: 5′-CCCTCACCATCATCCTCACT-3′<br>Reverse: 5′-TCCTTCGAGTGACAAACACG-3′
TGF-β1 primers	Forward: 5′-TGCGCTTGCAGAGATTAAAA-3′<br>Reverse: 5′-GCTGAATCGAAAGCCCTGTA-3′
TWEAK primers	Forward: 5′-CGAGCTATTGCAGCCCATTAT-3′<br>Reverse: 5′-ACCTGCTTGTGCTCCATCCT-3′
GAPDH primers	Forward: 5′-CTCATGACCACAGTCCATGC-3′<br>Reverse: 5′-ACACATTGGGGGTAGGAACA-3′

Abbreviations: CTGF, connective tissue growth factor; EGFR, epidermal growth factor receptor; Fn14, fibroblast growth factor inducible 14; IP-10, interferon gamma-induced protein 10; MCP-1, monocyte chemotactic protein 1; PDGFB, platelet-derived growth factor subunit B; RANTES, regulated on activation, normal T cell expressed and secreted; TGF, transforming growth factor; TWEAK, tumor necrosis factor-related weak inducer of apoptosis; GAPDH, glyceraldehyde 3-phosphate dehydrogenase

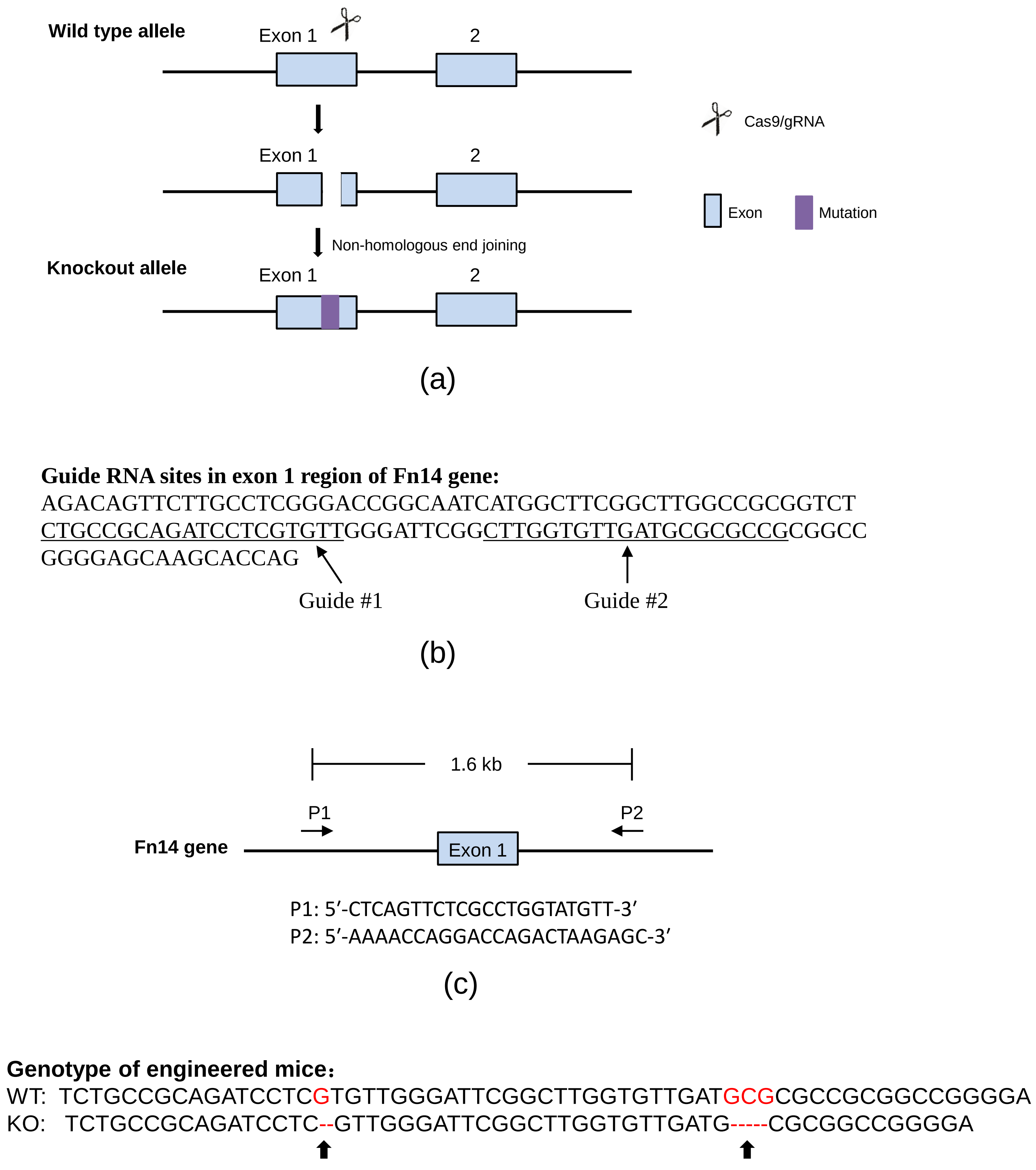

Figure S1: The strategy for generating Fn14 knockout mice by CRISPR/Cas9 method. (a) Cas9 mRNA and recombinant Cas9 protein are synthesized in vitro, and induce Fn14 gene mutation by non-homologous end joining. (b) Guide RNA is indicated in the exon 1 region of Fn14 gene. (c) By primers P1 and P2, Fn14 gene sequence is detected in engineered mice. (d) The genotype of engineered mice is analyzed. The mutated gene sites are indicated by arrows. CRISPR, clustered regularly interspaced short palindromic repeats; Cas9, CRISPR-associated 9; gRNA, guide RNA.

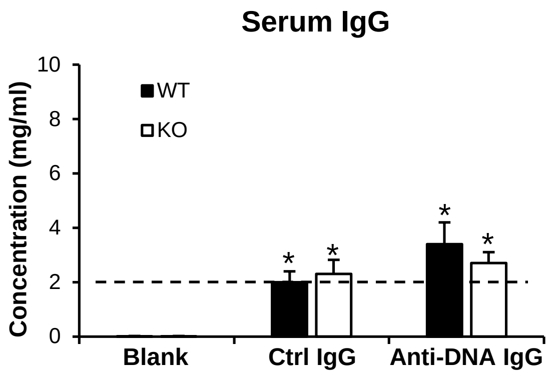

(a)

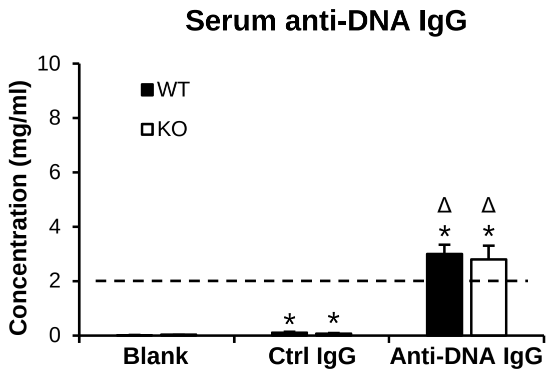

(b)

Figure S2: The serum levels of IgG are comparable between the WT and KO mice. These mice were injected with control IgG or anti-DNA IgG or as blank controls. Both total IgG (a) and anti-dsDNA IgG (b) were determined in serum samples. There were no significant differences between the WT and KO mice that received the same treatment. There were eight mice in each group. $^*p < 0.05$, compared with blank control group of the same strain; $\Delta p < 0.01$, compared with control IgG group of the same strain.
